# Pre-processing methods in chest X-ray image classification

**DOI:** 10.1371/journal.pone.0265949

**Published:** 2022-04-05

**Authors:** Agata Giełczyk, Anna Marciniak, Martyna Tarczewska, Zbigniew Lutowski

**Affiliations:** 1 Bydgoszcz University of Science and Technology, Bydgoszcz, Poland; 2 Faculty of Medicine Ludwik Rydygier Collegium Medicum in Bydgoszcz, Nicolaus Copernicus University in Torun, Bydgoszcz, Poland; University of Engineering and Technology Taxila Pakistan, PAKISTAN

## Abstract

**Background:**

The SARS-CoV-2 pandemic began in early 2020, paralyzing human life all over the world and threatening our security. Thus, the need for an effective, novel approach to diagnosing, preventing, and treating COVID-19 infections became paramount.

**Methods:**

This article proposes a machine learning-based method for the classification of chest X-ray images. We also examined some of the pre-processing methods such as thresholding, blurring, and histogram equalization.

**Results:**

We found the F1-score results rose to 97%, 96%, and 99% for the three analyzed classes: healthy, COVID-19, and pneumonia, respectively.

**Conclusion:**

Our research provides proof that machine learning can be used to support medics in chest X-ray classification and improving pre-processing leads to improvements in accuracy, precision, recall, and F1-scores.

## 1 Introduction

The occurrence of the COVID-19 pandemic in 2020 has shaken up the modern world. It has caused societies to close, crowded streets to become deserted, pubs and clubs to be silenced, and popular meeting places to die down. Currently, people all over the world are doing their best to overcome the pandemic’s impact on the social, medical, psychologic, economic, and industrial aspects of society. Currently, the main screening method for detecting COVID-19 infections is reverse transcriptase-polymerase chain reaction (RT-PCR) testing. The RT-PCR test can detect SARS-CoV-2 ribonucleic acid (RNA) from respiratory specimens (collected through nasopharyngeal or oropharyngeal swabs). In addition, patients suffering from COVID-19 can also present with abnormalities on chest X-ray images that are characteristic of infection [1]. This imaging modality is highly available and accessible in many clinical locations, and it is considered standard equipment in most healthcare systems. Moreover, CXR imaging is more widely available than CT imaging, especially in developing countries due to high equipment and maintenance costs. However, X-ray analysis can be time-consuming and requires highly educated specialists to interpret. But, the use of machine learning (ML)-based methods can improve efficiency, support medics in the diagnosis of COVID-19, speed up the time to diagnosis, and lighten the already burdened health care system.

At the same time, modern technologies have gathered more interest. Artificial intelligence (AI) and ML can be used in numerous applications such as cybersecurity [2], pedestrian detection [3], telemedicine [4], biometrics [5] or sports analytics [6]. Thus, the implementation of AI and ML in COVID-19 and other lung diseases seems to be the desired natural progression.

In this article, we present the impact pre-processing can have on the results of a classification system. We tested 5 different pre-processing methods and investigated their effect on the final classification. We conducted the study using a large public dataset. The proposed ML-based method was able to classify chest X-rays into 3 classes: normal (healthy), COVID-19, and pneumonia, which can be similar to images of patients infected by COVID-19.

## 2 Related work

As the COVID pandemic intensified, more investigators focused on automatic lung disease recognition. Milestones in pre-processing, feature extraction, and assigning a classification were required to achieve the required results. In addition, improvements were made at each step of the workflow.

The authors in [7] used a method based on U-NET and ResNet to perform the segmentation of CT images with an accuracy reaching 95%. The main obstacle in overcoming the segmentation problem is imperfect datasets. As mentioned in [8], medical image segmentation datasets suffer from scarce and weak annotations. In addition, acquiring the medical image’s data and annotations can be extremely difficult and expensive. In the article by [9], the authors proposed the use of a multi-level CNN-based preprocessor. The main reason for using this preprocessor was to dynamically enhance the lung regions that are useful in detecting COVID-19. Experiments using ReCoNet for differentiating COVID vs Pneumonia vs Normal were shown to have an accuracy > 97%. Authors in [10] proposed a novel, hybrid, multimodal deep learning system. With the use of Contrast-Limited Adaptive Histogram Equalization (CLAHE) and a Butterworth bandpass filter, the authors were able to enhance the contrast of X-ray images and eliminate the noise leading to an accuracy of 99.93%. The article by [11] highlights that pre-processing can improve a system’s accuracy. In this publication, the visibility of the diaphragm on the chest X-ray was mentioned. It was observed as a very light object in the bottom part of the chest. However, experiments using a convolutional neural network (CNN) reported improved results when the diaphragm was removed from the sample. In [12], an interesting and efficient approach based on the Bayes-SqueezeNet method was proposed. What is more, the authors described some details concerning data augmentation. In this specific study, the augmentation was performed offline using Gaussian blur, sheering, and decreasing/increasing brightness. The presented experiments provided promising results, namely an F1 score of 0.983. Mahdy et al. in [13], proposed a method to automatically classify COVID-19 chest X-rays using a multi-level threshold based on the Otsu algorithm and support vector machine (SVM). A SVM was also utilized in the article by [14]. In the presented approach, image enhancement was performed by increasing contrast, the Histogram of Oriented Gradients was used for features extraction, and Linear Regression and SVM were implemented for X-Ray classification resulting in an accuracy of 96%.

Models are using neural networks to analyze lung X-rays in cancer [15], pneumonia [16], and other lung diseases [17]. In the wake of the recent pandemic, deep learning methods have been used to analyze X-rays of patients potentially infected with the SARS-CoV-2 virus. A standard state-of-art approach using pre-trained CNNs was presented by authors in [18]. In the evaluation of AlexNet, VGG-16, VGG-19, SqueezeNet, GoogleNet, MobileNet-V2, ResNet-18, ResNet-50, ResNet-101, and Xception CNNs, the best results were achieved by Xception and ResNet-101, with an AUC of 0.994 for both networks and an accuracy of 99.02% and 99.51%, respectively. The authors in [19] connected pre-trained deep CNN models with various kernels SVM classifiers. This approach was compared with end-to-end training CNN models that performed worse. The best results (accuracy of 94.7%) were obtained by ResNet50 and SVM with linear kernels. Authors of [20], compared 3 deep-learning based CNN models, InceptionV3, Xception, and ResNeXt. Analysis was performed on 6,432 X-ray scans collected from the Kaggle repository. The highest accuracy was obtained for the Xception model (97.97%). Authors in [21] also tested InceptionV3 and compared the results with 8 more pre-trained CNNs. In that study, the overall accuracy for InceptionV3 was 54.41%, whereas the highest accuracy was achieved by the VGG16 model (95.88%). CoroDet, a novel CNN model, was developed by the authors of [22]. It allows X-ray images and CT scans to be classified into 2, 3, or 4 classes (COVID, Normal, non-COVID viral pneumonia, and non-COVID bacterial pneumonia) with an accuracy of 99.1%, 94.2%, and 91.2%, respectively.

As the COVID-19 pandemic started, researchers focused on providing datasets for performing scientific experiments. Selected datasets are listed in Table 1. As one can see, they contain not only COVID-19 chest X-ray images but also images of other lung diseases like pneumonia and SARS. In ML, the dataset must meet certain requirements. It should be representative of the disease and population being studied, consist of a large sample of data points, and be well balanced. Unfortunately, some of the listed datasets do not meet all of the above-mentioned requirements.

**Table 1:** The review of available X-ray datasets for COVID-19 classification.

Name	Classes and samples	Source
COVID-19 RADIOGRAPHY DATABASE	COVID—3616, Lung opacity—6012, Normal—10.2k, Viral pneumonia—1345	Kaggle
Covid19 Image Dataset	COVID—137, Normal—90, Viral pneumonia—90	Kaggle
Covid-19 X Ray 10000 Images	COVID—70, Normal—28	Kaggle
Chest X-ray (Covid-19 & Pneumonia)	COVID—576, Normal—1583, Pneumonia—4273	Kaggle
COVID19 Pneumonia Normal Chest Xray PA Dataset	COVID—2313, Normal—2313, Pneumonia—2313	Kaggle
Covid Chestxray Dataset	PA view—481, AP view—173, for over 15 different lung diseases	Github
Covid Patients Chest X-ray	COVID—162, Normal—162	Kaggle

## 3 Materials and methods

**Fig 1:**
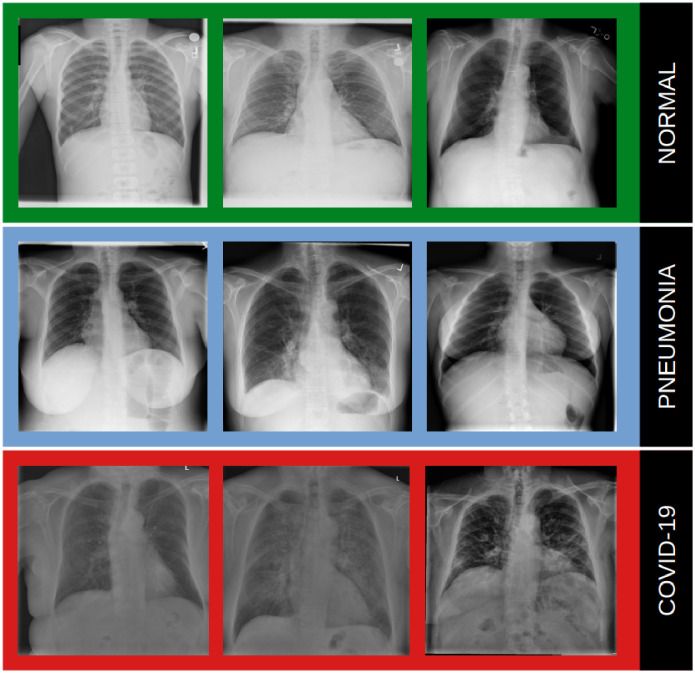
Examples of samples from the dataset: Normal (healthy), pneumonia and COVID-19.

In this research, we used the dataset available for the public at www.kaggle.com/amanullahasraf/covid19-pneumonia-normal-chest-xray-pa-dataset. It consists of images collected from the GitHub repository, Kaggle, Radiopedia, Italian Society of Radiology (SIRM), and Figshare data repository websites. The dataset was organized into 3 classes (COVID-19, pneumonia, and normal) containing posteroanterior (PA) chest X-ray images. A total of 6,939 samples were used in the experiment, and 2,313 samples were used for each class. Some examples of images used are presented in Fig 1.

**Fig 2:**
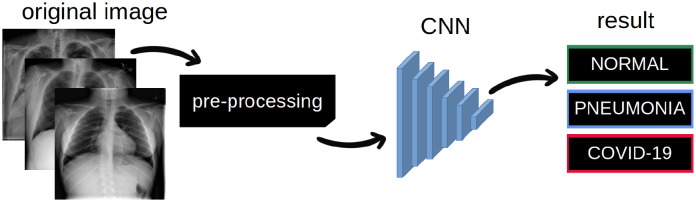
The overview of the proposed method.

To verify how the selected pre-processing method affects the final classification result, we proposed a baseline system. The general overview of this system is presented in Fig 2. The black box visible in Fig 2 marks the selected pre-processing method. The pre-processing step is an important element in the image analysis schema. It can enhance the original image and reduce noise or unwanted details. In our research, we examined 6 different approaches to pre-processing:

1. None—the baseline approach is not to use any method apart from size reduction.
2. Histogram equalization—this method extends the pixel’s intensity range from the original range to 0 to 255. Thus, the enhanced image has a wider range of intensity and slightly higher contrast.
3. Hist. eq. + Gaussian blur—this filter reduces some noise and unwanted details that can be confusing for the neural network; the filter kernel size was experimentally set to 5 × 5 size.
4. Hist. eq. + bilateral filter—this filter also reduces some noise and unwanted details that can be confusing for the neural network, but its main feature is to preserve edges; the experimentally set up parameters of the filter: *diameter* = 5, *σ*_*color*_ = *σ*_*space*_ = 75.
5. Adaptive masking—in [11] the authors proved that by removing the diaphragm from the sample it is possible to improve the classification results. In this proposed pre-processing method, we first found the maximum (max) and minimum (min) intensity of pixels and then applied the binary thresholding using the threshold expressed in Eq 1. The next step used morphologic closing. This creates the adaptive mask that after bitwise operation removes the diaphragm from the source image.
6. Adaptive masking + hist. eq. + Gaussian blur—this method joins adaptive masking with histogram equalization and Gaussian blur.
  $$\begin{array}{c} threshold=min+0.9\cdot (max-min) \end{array}$$ (1)

At image classification, a CNN was implemented. The CNN model can provide human-like accuracy in classifying various images (Fig 2). A convolution network can be described as a chain of convolution layers, with rectified linear unit activation functions, pooling layers, and batch normalization operations. The architecture of a CNN is analogous to that of the connectivity pattern of neurons in the human brain. In fact, it was inspired by the organization of the visual cortex. Individual neurons respond to stimuli only in a restricted region of the visual field known as the receptive field. A collection of these fields overlap to encompass the entire visual area. The hierarchical network provides high-level feature maps, reduced computation complexity, and improved generalization ability. The advantages of CNNs have led to their wide implementation in image processing. For chest X-ray analysis, CNN was implemented in [23–27]. The neural network used in this paper consists of 12 layers:

- Conv2D—it is a convolution layer with 64 filters with dimensions of 3 × 3 and the activation function ReLU, the dimensions of the input data is also introduced
- MaxPooling with a size of 2 × 2
- Conv2D—it is a convolution layer with 64 filters with dimensions of 3 × 3 and the activation function ReLU
- MaxPooling with a size of 2x2
- Conv2D—it is a convolution layer with 128 filters with dimensions of 3 × 3 and the activation function ReLU
- MaxPooling with a size of 2x2
- Conv2D—it is a convolution layer with 128 filters with dimensions of 3 × 3 and the activation function ReLU
- MaxPooling with a size of 2x2
- Flatten—it is a data flattening layer, it has no additional parameters
- Dropout layer—randomly sets input units to 0 with a frequency rate at each step during training time, which helps prevent overfitting. Inputs not set to 0 are scaled up by 1/(1—rate) such that the sum over all inputs are unchanged, in this case, the rate equals 0.2
- Dense layer—in which each neuron is connected to each neuron of the previous layer with the unit parameters (positive integer, dimensionality of the output space) equal to 512, with the activation function ReLU
- Dense—with unit parameters equal to 3, with softmax activation function.

The output from the neural network shows the probability of an image belonging to one of the three classes thanks to the softmax activation function in the last layer. The network selects the classification with the highest probability and identifies it as the final result. All the experiments were executed using the online Kaggle notebook. There were almost 7,000 samples in the dataset. We decided to divide the dataset into three disjoint subsets: training-65%, validating-15%, and testing-20%. All of the experiments were executed 3 times to prove their independence from the learning data. Due to a balanced dataset, we did not need any sample augmentation.

## 4 Results

**Fig 3:**
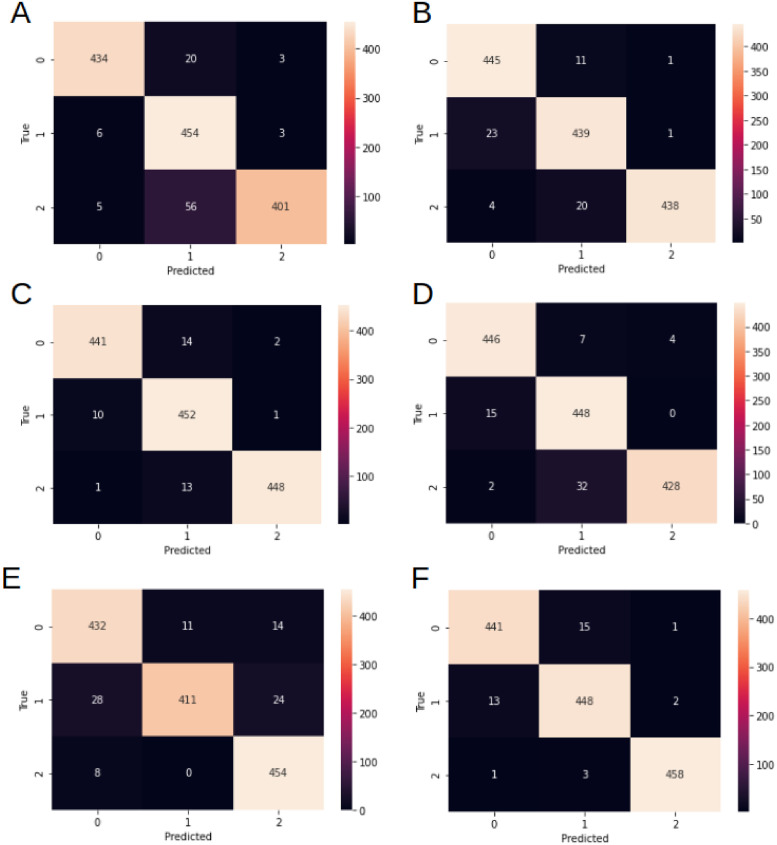
Confusion matrices. Confusion matrices for the experiments varying by the pre-processing method—A: none, B: histogram equalization, C: histogram equalization + Gaussian blur, D: histogram equalization + bilateral filter, E: adaptive mask, F: adaptive mask + histogram equalization + Gausssian blur.

The above-mentioned experiments provided some promising results. We used 4 parameters for the evaluation methods—accuracy, precision, recall, and F1-score. The parameters were calculated using a confusion matrix (presented in Fig 3) reporting the number of true positives (TP), true negatives (TN), false positives (FP), and false negatives (FN). The evaluation parameters were calculated using Eqs 2–5.

$$\begin{array}{c} Accuracy=\frac{TP+TN}{TP+TN+FP+FN} \end{array}$$ (2)

$$\begin{array}{c} Precision=\frac{TP}{TP+FP} \end{array}$$ (3)

$$\begin{array}{c} Recall=\frac{TP}{TP+FN} \end{array}$$ (4)

$$\begin{array}{c} F1-score=2\cdot \frac{precision\cdot recall}{precision+recall} \end{array}$$ (5)

The results without any additional pre-processing resulted in an accuracy of 93% and a F1-score in the range of 91% to 96% for the three evaluated classes. Introducing a pre-processing method improved the parameters, for instance, applying histogram equalization raised the precision, recall, and F1-score by 2%. Nevertheless, the most promising approach was joining histogram equalization with Gaussian blurring and adaptive masking. This approach ensured all evaluated parameters exceeded 97%. The results for each pre-processing method are presented in Table 2.

**Table 2:** Obtained results for different pre-processing methods: 1—none, 2—histogram equalization, 3—Gaussian blur + hist. equalization, 4—bilateral filter + hist. equalization, 5—adaptive masking, and 6—adaptive masking + Gauss. blur + hist. eq.

Method	Class	Accuracy	Precision	Recall	F1-score
1	Normal	0.9754	0.9753	0.9497	0.9623
	COVID-19	0.9385	0.8566	0.9806	0.9144
	Pneumonia	0.9515	0.9853	0.8680	0.9229
	Average	0.9551	0.9390	0.9327	0.9332
2	Normal	0.9712	0.9428	0.9737	0.9580
	COVID-19	0.9602	0.9340	0.9482	0.9411
	Pneumonia	0.9812	0.9955	0.9481	0.9712
	Average	0.9711	0.9574	0.9567	0.9567
3	Normal	0.9805	0.9757	0.9650	0.9703
	COVID-19	0.9725	0.9436	0.9762	0.9597
	Pneumonia	0.9877	0.9933	0.9697	0.9814
	Average	0.9802	0.9709	0.9703	0.9704
4	Normal	0.9566	0.9633	0.9759	0.9696
	COVID-19	0.9609	0.9199	0.9676	0.9432
	Pneumonia	0.9725	0.9907	0.9264	0.9575
	Average	0.9566	0.9580	0.9566	0.9567
5	Normal	0.9559	0.9231	0.9453	0.9341
	COVID-19	0.9544	0.9739	0.8877	0.9288
	Pneumonia	0.9667	0.9228	0.9827	0.9518
	Average	0.9590	0.9399	0.9386	0.9382
**6**	**Normal**	**0.9782**	**0.9692**	**0.9650**	**0.9671**
	**COVID-19**	**0.9761**	**0.9614**	**0.9676**	**0.9645**
	**Pneumonia**	**0.9949**	**0.9935**	**0.9913**	**0.9924**
	**Average**	**0.9831**	**0.9747**	**0.9746**	**0.9747**

## 5 Discussion

### 5.1 Threads to validity

The presented method is very powerful in diagnosing COVID-19 and pneumonia. However, there are some issues to keep in mind if this modality is implemented in patient care. The first issue is responsibility—who is going to be responsible for the ML-based decision? Thus, we have to specify that the proposed method is not a tool for replacing the educated specialist but to improve his/her work and support the diagnostic process. Furthermore, to implement the proposed method, the explainability of the module must be added to the described pipeline. Explanation of the decisions would be the main task for such a module. It would give the reasons why the sample was classified to a specific class and would be very helpful in marking the part of the image responsible for the decision. A few explainable ML-based methods have been published recently (see: [28–30]).

The second issue is the quality of images used in the learning process. In the presented approach, the dataset was obtained from numerous sources: Github, Kaggle, Radiopedia, SIRM, and Figshare data repository websites. We deeply trust that the images provided are labeled correctly and submitted by an expert. The labeling process seems to be the most challenging, expensive, and time-consuming part of the chest X-ray image analysis system. Due to the relatively recent identification of COVID-19, the number of samples in the datasets are limited. Some commercial projects are working on gathering COVID-19 data (including chest X-rays), but these datasets are currently not available to the public.

### 5.2 Comparison to SOTA

The detection of COVID-19 and other lung diseases using chest X-ray imaging has recently been widely investigated. Table 3 provides detailed results from the current literature. Unfortunately, not all authors evaluate the same set of parameters as in our study, namely: Accuracy, Precision, Recall, and F1-score. Even though, the provided results prove that our solution is comparable to other SOTA methods.

**Table 3:** Comparison between the proposed method and SOTA methods.

Reference	Dataset	Result
**proposed**	**Kaggle**	**Acc. = 98.31%, Prec. = 97.47%, Rec. = 97.46%, F1 = 97.47%**
Ahmed et. al. [9]	GitHub	Acc. = 97.48%, Prec. = 97.39%, Spec. = 97.53%, MCC = 92.49%
Al-Waisy et. al. [10]	Github, Kaggle	Acc. = 99.93%, Prec. = 100%, Rec. = 99.90%, F1 = 99.93%
Mahdy et. al. [13]	GitHub	Acc. = 97.48%, Prec. = 95.276%, Spec. = 99.7%
Ucar et. al. [12]	arXiv, Kaggle	Acc. = 98.3%, Prec. = 98.3%, Rec. = 98.3%, F1 = 98.3%

## 6 Conclusions

COVID-19 is a highly infectious disease caused by the most recently discovered coronavirus and is considered a pandemic according to the World Health Organization. Even though vaccines were introduced at the beginning of 2021, there is a strong need for fast and accurate tools to improve the efficiency of the healthcare system.

In this article, we proposed a novel approach for the fully automated analysis of COVID-19 chest X-ray images using a neural network. Our approach was successful in distinguishing images into three classes: COVID-19, pneumonia, and normal (healthy). We also presented an improvement in the proposed method, namely the pre-processing part of the ML-based system. In this early step of image analysis, a few crucial operations are performed: adaptive masking (the part of the image that is very light is removed), histogram equalization, and Gaussian blur (removes noise and some unwanted details). We proved that the proposed pre-processing method increases the efficiency of the system as the F1-score raised from 93% to over 97%. Our results are comparable to other similar ML-based approaches in the literature, but there are plenty of pre-processing methods that can improve the efficiency of the system and be implemented in future work.
